# Storybooks aren't just for fun: narrative and non-narrative picture books foster equal amounts of generic language during mother-toddler book sharing

**DOI:** 10.3389/fpsyg.2014.00325

**Published:** 2014-04-16

**Authors:** Angela Nyhout, Daniela K. O'Neill

**Affiliations:** Department of Psychology, University of WaterlooWaterloo, ON, Canada

**Keywords:** generic language, parent-child interactions, book sharing, narrative books, informational books, book genre, contextual influences

## Abstract

Parents and children encounter a variety of animals and objects in the early picture books they share, but little is known about how the context in which these entities are presented influences talk about them. The present study investigated how the presence or absence of a visual narrative context influences mothers' tendency to refer to animals as individual characters or as members of a kind when sharing picture books with their toddlers (mean age 21.3 months). Mother-child dyads shared both a narrative and a non-narrative book, each featuring six animals and matched in terms of length and quantity of text. Mothers made more specific (individual-referring) statements about animals in the narrative books, whereas they provided more labels for animals in the non-narrative books. But, of most interest, the frequency and proportion of mothers' use of generic (kind-referring) utterances did not differ across the two different types of books. Further coding of the content of the utterances revealed that mothers provided more story-specific descriptions of states and actions of the animals when sharing narrative books and more physical descriptions of animals when sharing non-narrative books. However, the two books did not differ in terms of their elicitation of natural facts about the animals. Overall, although the two types of books encouraged different types of talk from mothers, they stimulated generic language and talk about natural facts to an equal degree. Implications for learning from picture storybooks and book genre selection in classrooms and home reading are discussed.

## Introduction

Individuals possess knowledge about events, objects, and living things that they have not observed first hand. Historical events, entities too distant, and those too minute, are beyond what the average human can observe. Yet, most of us possess at least some basic information about the ice age, the planet Jupiter, and atoms. Given that most individuals will never have the opportunity to participate in an archeological dig or peer through an electron microscope, we must learn about such entities indirectly. Two important sources of this information for children, relevant to the present article, are the testimony of other individuals (Harris and Koenig, 2006) and picture books (DeTemple and Snow, 2003).

As children encounter objects (e.g., a bicycle) and animals (e.g., an elephant) in picture books and other settings, they may take them to be individuals (e.g., Jamie's bicycle; Babar) or members of a category (e.g., bicycles; elephants) (Hall et al., 2001). When referring to entities in the world, certain linguistic markers can distinguish whether one is communicating about the entity as an individual or as a member of a category. Information that is conveyed using specific language tends to refer to individuals (e.g., “Shadow has a soft coat”), whereas information that is conveyed using generic language pertains to categories (e.g., “Dogs have four legs”). Information that is delivered using generic language is readily incorporated into children's knowledge-bases (Prasada, 2000; Cimpian and Markman, 2008). This can lead to robust learning of facts about the world (e.g., elephants are very social animals) that is resistant to counter-examples (e.g., Rowan the elephant isn't sociable). Thus, whether children consider an entity as an individual or as a member of a category can influence whether they incorporate the information they encounter into their knowledge-base. As such, it is of interest to investigate the factors that influence whether talk about entities is primarily individual-referring vs. category-referring.

### Contextual influences on generic language use

In a study by Gelman et al. (2005), parent-child conversations included more generic utterances when dyads interacted with pictures of objects than when they interacted with the objects themselves. Gelman and colleagues suggested this was because pictures are more representational of categories, whereas objects are more likely to be perceived as individuals in their own right. Comparing parent-toddler conversations across picture book sharing and toy play, Gelman and Tardif (1998) similarly found that generic utterances were more common during picture book sharing than during toy play, comprising 4.7% of utterances during book sharing. Thus, it is clear that contextual factors influence the use of generic, category-referring language during interactions between parents and their preschoolers or toddlers. In subsequent studies, Gelman et al. (2013) found that adults and 5- and 6-year-olds used more generics when they were in a pedagogical context or role than when they were in a “peer-to-peer” situation. These findings provide support for Csibra and Gergely's (2009) theory of natural pedagogy, which posits that pedagogical contexts encourage the communication of generic information.

Given that picture book sharing seems to be a particularly fruitful setting for talk about categories, it is of interest whether the format in which animals are presented in books can influence the use of language referring to individuals and categories. Recently, in a first study to investigate this, Gelman et al. (2013) analyzed 75 informational and narrative picture books designed for 4- to 9-year-olds and found that informational books contained significantly more generic noun phrases than narrative books. Both types of books, however, contained significantly more specific noun phrases than they did generics.

Together, these studies established that context does indeed influence the extent to which entities are considered as individuals or as members of a category, as reflected by the relative frequency of generic language use. Moreover, two of these studies (Gelman and Tardif, 1998; Gelman et al., 2005) demonstrated that generic language is present in mothers' talk with their toddlers (25- to 38-month-olds and 19- to 23-month-olds, respectively), the population relevant to the present study (18- to 25-month-olds).

Given the young age of the children in the present study, another important consideration is the extent to which children of this age are sensitive to cues in language that distinguish generic and specific referents. Gelman and Raman (2003) found that 2-year-olds were sensitive to relevant morphosyntactic cues, such as the presence or absence of the definite article, *the* (e.g., “Do the birds fly?” vs. “Do birds fly?”). Graham et al. (2011) found that 30-month-olds, but not 24-month-olds were able to distinguish between generic and non-generic utterances to make inferences about novel kinds. However, because this task required the extra step of inductive inference, the findings do not necessarily indicate a lack of comprehension amongst the younger age group. Although there is currently no evidence that children are able to distinguish between such syntactic cues before the age of two, the presence of such syntactic distinctions in the input from adults certainly precedes their understanding.

### The effect of book genre

As mentioned above, picture book sharing appears to stimulate generic language use more than other types of parent-child interactions that have been studied. How parents' generic language use may vary as a function of book genre has not, to our knowledge, been a subject of previous investigation. In particular, because the educational value of storybooks has been called into question (e.g., Torr and Clugston, 1999; Bosman, 2010), it is of interest to investigate the extent to which different types of books for young children encourage parents to express generic knowledge and facts.

Recent media reports suggest that picture storybook sales have declined as parents seek books that they believe are more educational (e.g., Bosman, 2010), such as early readers and informational books. Non-narrative books (e.g., books focusing on building vocabulary) for children frequently make claims about the types of skills and knowledge they can provide, whereas storybooks tend to provide only short synopses. Whether the nature of the communicative interaction that arises is indeed more ‘educational’ when sharing non-narrative books, compared to narrative books, is relatively unstudied (Nyhout and O'Neill, 2013). Because of documented cross-contextual differences in parents' talk, it is of interest to investigate how the genre of book parents share with their children may affect their use of more “educational” or “pedagogical” talk. Although pedagogical contexts certainly encourage many types of language, our focus in the present study was specifically on two types of language as indices of *pedagogical language*: (1) generic language and (2) natural facts.

Most investigations of the influence of book genre have compared parents' abstract comments and questions during narrative and informational (i.e., non-fiction) book sharing interactions between parents and *preschoolers*. Generally, these studies have found that mothers' talk is more abstract during informational book sharing than during narrative book sharing (Torr and Clugston, 1999; DeTemple, 2001; Anderson et al., 2004; Price et al., 2009).

In contrast to findings with preschoolers, we found that mothers' talk with *toddlers* was more complex during narrative book sharing than during non-narrative book sharing (Nyhout and O'Neill, 2013). This different pattern of results was likely due to differences in the age of the children and the fact that we employed greater experimental control over the books being compared. Previous studies comparing narrative and informational books used books that differed on a number of dimensions such as subject, number of pages, and quantity of text (Torr and Clugston, 1999; DeTemple, 2001; Anderson et al., 2004).

In addition to this sizable body of work investigating differences in parents' talk across book genres, in one study (Ganea et al., 2011) it was also noted that children were able to generalize a principle (camouflage) learned from a picture book to real world problems. They were able to do this regardless of whether the information was presented in a factual or intentional (i.e., narrative) framework, although the specific focus of the study was not on book genre.

### Rationale for the present study

In the present study, we analyzed the interactions from Nyhout and O'Neill (2013), but in this case were interested in whether the context in which an animal was presented in a picture book influenced the extent to which mothers referred to the animal as an individual or as a member of a kind. We were specifically interested in whether the *presence or absence of narrative context* influenced mothers' tendency to refer to the characters as individuals or as members of a kind.

Suppose that a book features 6 animals. One prediction might be that presenting the 6 animals in the framework of a narrative will lead parents and children to consider the animals as *individuals* (e.g., The Bear). Indeed, it is often the intention of tellers of narratives to introduce the audience to individuals; the presence of characters is at the core of what it is to be a narrative (Bruner, 1986). In contrast, if the 6 animals are presented in a contextless (i.e., non-narrative) manner typical of didactic books, parents and children may be more likely to think about and discuss *categories or kinds* of animals (e.g., bears). Indeed, introducing the audience to categories of animals or objects is often the goal of creators of non-narrative books (Martin, 1985). In support of these views, the content analysis of the texts of children's narrative and informational picture books by Gelman et al. (2013), discussed above, did find that the texts in informational books contained significantly more generic noun phrases than the texts of narrative books.

But in book sharing interactions between parents and children, parents' talk may be differentially influenced by book genre. This may especially be the case in parent-toddler interactions, given that books with little to no text may be shared, and parents are freer to talk about the content as they wish, as found in Nyhout and O'Neill (2013). Thus, with respect to the use of generic or non-generic language, it is not necessarily the case that differences found to exist within the *texts* of different genres of books will pertain in the same manner to parents' *talk* when sharing these different genres of books with their child. Indeed, in a broader context, the syntactic constructions presented in picture books texts for 2-year-olds have been found to significantly differ from those occurring in parents' talk with children aged 21–32 months (Cameron-Faulkner and Noble, 2013). It is an open question whether findings on genre differences in book texts extend to parents' talk, especially when sharing books with very young children, who are the focus of interest in our present study.

Thus, to explore the influence of book genre on talk about individuals and kinds, we compared mothers' talk with their toddlers while they shared two picture books, each about 6 animals: a short narrative and a non-narrative book. In the narrative books, the animals were introduced one-by-one within the context of a story with background scenes and with no text except for the label of each animal when first introduced. In the non-narrative books, the animals were introduced one-by-one alone on a blank page with a single label (see Methods). Thus, the key manipulation was the presence or absence of a narrative context.

We were interested in both the *framing* and the *content* of mothers' utterances. In coding the framing, we looked at whether mother's statements were presented with a *generic* (e.g., “*Lions* say “roar””) or *specific* subject (e.g., “*He* says “roar””), or as a label (e.g., “That's a lion”), based on the coding scheme developed by Gelman et al. (2005). In coding the content, we looked at whether mothers' utterances comprised a *physical description* of an animal in the book (e.g., “He has black and white fur”); a *story-specific* description of an animal's state or action (e.g., “The bear is sleepy”), or a *natural fact*, which included a description of an unobservable behavior or property of an animal in the book (e.g., “He (badger) uses his sharp claws and goes dig, dig, dig”). Note that these two levels of coding (framing and content) can offer unique information, especially when the content concerns physical descriptions or natural facts. For example, a *natural fact* could be presented by a mother using generic (e.g., “Armadillos can curl up in balls”) or specific syntax (e.g., “He can curl up in a ball”).

It seems reasonable to expect that mothers will employ more specific utterances during narrative book sharing; that is, as the animals engage in actions unique to the story, it is likely mothers will describe these actions (e.g., “The elephant is escaping from the cage!”). However, the differences across narrative and non-narrative genres in generic language use are harder to predict, if differences are present at all. On the one hand, one may predict that the non-narrative books will elicit more generic language from mothers, because the animals in the non-narrative books may be seen as more representational of categories than when they are presented in the framework of a narrative. Note that this prediction would be in line with the predictions and findings of Gelman et al. (Gelman and Tardif, 1998; Gelman et al., 2005). On the other hand, one may predict that the narrative books will elicit more generic language from mothers, because the context provided by the narrative books may trigger generic knowledge about the animals. For example, reading the popular children's book *Stellaluna* may remind parents of facts about bats' diet, habitat, and behaviors in ways that a picture of a bat may not. Thus, we did not have firm predictions about how narrative context would influence mothers' use of generic language, though we expected more specific language during narrative book sharing.

## Methods

### Participants

Twenty-five mother-toddler dyads participated in the study (12 girls, mean child age = 21.3 months, range = 18.9 to 25.4 months). Two additional dyads participated but were dropped because they failed to complete one of the books (*n* = 1) and because the child was distracted for most of the book sharing interaction (*n* = 1). Participants were recruited from a university laboratory database of local families and through advertisements in the community. Fifteen mothers (63%) had completed an undergraduate degree or higher, eight had completed a technical college diploma (33%), and two had completed a high school diploma (8%). Mothers ranged in age from 25 to 39 (mean = 32.6 years). Dyads were screened for prior exposure to the two books used in the study at the time of recruitment.

### Materials and design

The books for the study were created by adapting two commercially-available children's picture books, *Good Night, Gorilla* (Rathmann, 1994) and *Don't Wake Up the Bear!* (Murray, 2003). In *Good Night, Gorilla*, a zoo keeper makes his rounds to ensure all the animals are locked away for the night. Unbeknownst to the zoo keeper, a gorilla has stolen his keys and sneaks behind him as he walks through the zoo and unlocks the other animals' cages. In turn, an elephant, a lion, a giraffe, a hyena, and an armadillo all escape. The animals then follow the zoo keeper back to his house. In *Don't Wake Up the Bear!*, we are introduced to a bear sleeping in his cave in a snowy forest. Five other woodland animals, who are out in the cold, are trying to find a warm place to sleep. One-by-one, a hare, a badger, a fox, a squirrel, and a mouse come by until they are all cuddled up together in the bear's cave. Both original stories continue, but we ended the adapted versions at the points described to allow for consistent length across book versions (described below). Both books contained six animals in total and began with a single animal that was joined by a new animal on each page, until all six were together.

From each original book, we created one narrative and one non-narrative version for our study, which were matched for length in terms of both number of pages and amount of text, and the target content of interest (i.e., the six animals). *Good Night, Gorilla* and *Don't Wake Up the Bear!* were renamed *Animals at the Zoo* and *Animals in the Woods*, respectively. The narrative versions of each book included the same original illustrations, but the text on each page was removed and replaced with a single label per page identifying the focal animal. For the non-narrative versions of each book, we cropped the focal animal from each page in the original and placed it in the center of a blank page with the same single label. Thus, the two versions of each book included the same text (the label for each animal) and the same focal animal. Critically, the illustration of the animal was the same across both genres. The manipulation of interest was therefore the presence or absence of an illustrated narrative context. For both the narrative and non-narrative versions of each book, the final page presented all animals together with no text. The narrative versions included the original illustrations, whereas the non-narrative versions included discrete illustrations of each animal that were arranged in a line across the page. Readers interested in seeing the two versions of the books may contact the authors for a copy.

Our design was within-subjects, and dyads shared either the narrative version of *Animals at the Zoo* and the non-narrative version of *Animals in the Woods*, or the narrative version of *Animals in the Woods* and the non-narrative version of *Animals at the Zoo*. The order of presentation was fully counterbalanced.

### Procedure

Dyads were presented with the first book and were asked to share the book as they would at home. The second book was placed in a box, outside the child's view, and mothers were asked to retrieve it after finishing the first book. They were asked to share each book only once, from front to back. Dyads sat with the child on the mother's lap, in separate chairs beside each other, or on the floor together. The interactions were video recorded.

### Transcript coding

The interactions were transcribed using the Codes for the Human Analysis of Transcriptions (CHAT) transcription system (MacWhinney and Snow, 1990; MacWhinney, 2000). Because each dyad shared two books (one narrative and one non-narrative), there were two transcripts per dyad.

To begin with, all utterances in the transcripts that referred to one of the six animals in each book (i.e., that had an animal as their subject, except in the case of labeling) were selected as utterances to be coded. Utterances that referred to aspects of the background scene (e.g., “Look at that moon”) and events in the child's life (e.g., “Remember when we saw a lion at the zoo?”) were not included in the coding.

#### Utterance framing: generic subject, specific subject, or labels

The utterance framing coding scheme was adapted from Gelman et al. (2005), who coded for generic phrases, individuating phrases, and ostensive labeling phrases. Under the Gelman et al. (2005) coding scheme, generic phrases included those with bare plurals (e.g., elephants), indefinite singulars (e.g., an elephant), and definite singulars (e.g., the elephant) as their subject. Specific (individuating) phrases included those with proper names (e.g., Babar), singular pronouns (e.g., he/she), and count nouns (e.g., some elephants) as their subject. Given that the picture books used in this study only included labels for the animals and not proper names (e.g., Babar), parents' commonly referred to specific animals using definite singulars (e.g., The elephant is sleeping.). Thus, such definite singular constructions were coded as specific in all cases. Labeling phrases were those that served to place an individual in a category (e.g., “that's a lion”) and did not contain any additional descriptive information (e.g., “that's a funny-looking hyena”).

#### Utterance content: physical descriptions, natural facts, and story-specific utterances

In an initial look at our transcripts, we noticed that mothers would often make kind-relevant statements using a specific subject (e.g., a singular pronoun: “he says roar”). Thus, all the generic and specific subject utterances identified were further coded with respect to *utterance content*. In particular, each utterance's content was coded as either physical description, story-specific, or natural fact. Table 1 provides more detailed descriptions and examples of the coding of utterance content. Via this coding, we sought to determine whether the two picture book genres differed in the extent to which they stimulated talk about the animals that provided unobservable natural facts vs. information about observable characteristics (physical descriptions) and depicted states and events (story-specific). Our primary interest was in natural facts, because it is this type of information that is often expected to be conveyed using generic language.

**Table 1:** **Description and examples of the three subcategories for the utterance framing and utterance content categories**.

**Coding category**	**Subcategory**	**Description**	**Examples**
Utterance framing	Generic	Utterances that include bare plurals or indefinite singulars as their subjects	“Squirrels like to climb trees”
			“A hyena looks like a dog”
	Specific	Utterances that include definite singulars^*^, proper names, singular pronouns, or count nouns as their subjects	“The bear is sleeping”
			“He's getting out of his cage”
	Labels	Utterances that designate a particular animal as a member of a kind	“Now, this is a hare”
			“He's a gorilla”
Utterance content^**^	Physical description	Utterances that describe an observable, physical property of the animal	“Giraffe has a long neck”
			“A badger is black and white”
	Story-specific	Utterances that describe a specific action or state of an animal in the story	“The gorilla is unlocking the cage!”
			“The bear is sleepy”
	Natural fact	Utterances that describe an unobservable property of the animal. These included utterances that classify the animal, or provide information about the animal's habitat, behavior (e.g., animal sounds), or diet	“He's a type of ape”
			“The hyena says (makes laughing noise)”

* As described on p. 11, given the nature of the books used, definite singular constructions were more appropriately coded as instances of specific framing rather than generic framing, in contrast to Gelman et al. (2005).

** Labels were not included in this level of coding.

Two coders, one blind to the purpose of the study, coded the utterances that referred to animals, as described above, in all 50 transcripts. Coding agreement was excellent for both utterance framing (κ = 0.90) and content (κ = 0.96).

## Results

Overall, as reported in Nyhout and O'Neill (2013), mothers produced an average of 50.40 (*SD* = 30.36) utterances during narrative book sharing and 35.56 (*SD* = 14.58) utterances during non-narrative book sharing, *t*_(24)_ = 2.90 *p* = 0.008. Note that the greater quantity of talk during narrative book sharing can mostly be attributed to the presence of the background scene, which was present in the narrative versions, but not the non-narrative versions of each book. Mothers had the opportunity to discuss aspects of the background scene during narrative book sharing (e.g., a snowy tree, the animals' cages). Because mothers talked significantly more during narrative book sharing, Nyhout and O'Neill (2013) analyzed results for both frequency and proportion, as we will also report here with respect to the proportion of total maternal utterances (see Table 2). In most cases, the proportion results paralleled the frequency results. As such, we only present proportion results when they deviate from the frequency results in terms of patterns of significance. In cases of violations of the assumption of sphericity, we made Greenhouse-Geisser adjustments to the degrees of freedom. Because many of the children in the study produced only single-word utterances, or utterances that were unintelligible, we present only data on mothers' utterances. There were no significant effects of book version (e.g., narrative *Zoo* vs. narrative *Woods)*, order of book presentation, or child gender, so all results are analyzed together.

**Table 2:** **Mean (SD) frequency and proportion of utterances for each utterance framing and content type for narrative and non-narrative books**.

	**Generic**		**Specific**		**Label**		**Physical description**		**Story-specific**		**Natural fact**	
	**Narr**	**NonN**	**Narr**	**NonN**	**Narr**	**NonN**	**Narr**	**NonN**	**Narr**	**NonN**	**Narr**	**NonN**
Frequency	1.68	1.56	8.72^a^	5.64^b^	7.84	9.32	0.76^a^	2.72^b^	7.52^a^	1.64^b^	2.16	2.84
	(2.02)	(1.33)	(5.03)	(4.13)	(5.01)	(3.59)	(0.97)	(2.81)	(4.79)	(2.00)	(2.12)	(1.89)
Proportion: total maternal	0.04	0.05	0.19	0.16	0.18^a^	0.31^b^	0.02^a^	0.07^b^	0.16^a^	0.05^b^	0.05	0.09
	(0.06)	(0.05)	(0.10)	(0.13)	(0.09)	(0.20)	(0.02)	(0.08)	(0.10)	(0.06)	(0.06)	(0.06)

Means with different superscripts for narrative and non-narrative within each category were significantly different at p < 0.0125.

### Utterance framing

To investigate our question of whether book genre influenced the framing of utterances mothers used, we conducted 2 × 3 repeated-measures analyses of variance (ANOVA) with book genre (narrative or non-narrative) and utterance framing type (generic, specific, or labeling) as within-subjects factors for both frequency and proportion data.

#### Frequency

Results of the frequency ANOVA demonstrated that there was no significant main effect of genre: the narrative (*M* = 18.24, *SD* = 8.15) and non-narrative genres (*M* = 16.52, *SD* = 5.44) did not differ in terms of the average number of animal-referring utterances they elicited, *F*_(1, 24)_ = 1.46, *p* = 0.238, η^2^_*p*_ = 0.06. There was, however, a difference between the three utterance framing types when combined across genre, *F*_(1.49, 35.74)_ = 40.94, *p* < 0.001, η^2^_*p*_ = 0.63. To explore this difference in further detail, we conducted *post-hoc t*-tests with a Bonferroni-corrected α-value of 0.0125, as was the case with all other *post-hoc* tests described below. Collapsing across genre, the frequency of generic statements (*M* = 1.62, *SD* = 1.29) was significantly lower than the frequency of labels (*M* = 8.58, *SD* = 3.37), *t*_(24)_ = 11.38, *p* < 0.001, and the frequency of specific statements (*M* = 7.18, *SD* = 3.91), *t*_(24)_ = 7.29, *p* < 0.001. The frequencies of labels and specific statements did not differ significantly, *p* = 0.181.

**Figure 1:**
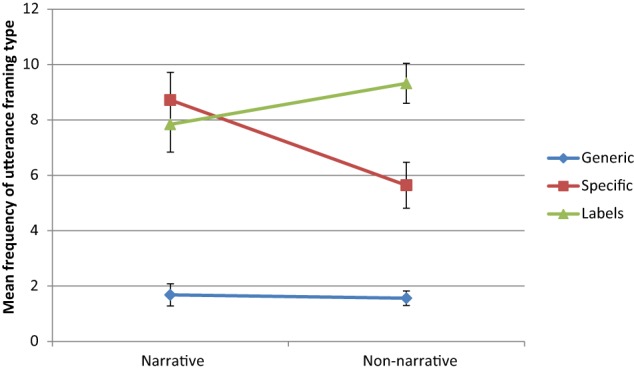
**Mean frequency (±SE) of generic, specific, and labeling statements across narrative and non-narrative book sharing**.

Turning to the main results of interest, we found that the genre by utterance framing type interaction was significant, *F*_(2, 48)_ = 6.36, *p* = 0.004, η^2^_*p*_ = 0.21. Figure 1 displays the results of this interaction. The frequency of *generic statements* did not differ significantly between narrative and non-narrative book sharing, *p* = 0.791. The frequency of *labels* also did not differ significantly between narrative and non-narrative book sharing, *p* = 0.192. The frequency of *specific statements* was significantly greater during narrative book sharing than during non-narrative book sharing, *t*_(24)_ = 5.08, *p* = 0.004. See Table 2 for a display of the means of frequency and proportion interactions.

#### Proportion

The patterns of significance for the main effects and interaction echoed those for the frequency analysis. Two differences were however found with respect to the *post-hoc t*-tests for the interaction. None of these differences concerned generic statements. First, recall that when comparing frequency, the two genres did not differ in terms of the mean number of labels they elicited from mothers. However, labeling was significantly greater during non-narrative book sharing than during narrative book sharing when analyzed as a proportion of total maternal utterances. Second, recall that there were significantly more specific statements during narrative book sharing than during non-narrative book sharing. This difference did not hold when compared as a proportion of *total* utterances (*p* = 0.193).

#### Utterance content

To investigate our question of whether book genre influenced the content of mothers' utterances, we conducted 2 × 3 repeated-measures analyses of variance (ANOVA) with book genre (narrative or non-narrative) and content (physical description, natural fact, or story-specific) as within-subjects factors for both frequency and proportion data.

#### Frequency

Of the generic and specific utterances coded further for content, significantly more occurred during narrative book sharing (*M* = 3.48, *SD* = 1.92) than during non-narrative book sharing (*M* = 2.40, *SD* = 1.61), *F*_(1, 24)_ = 7.42, *p* = 0.012, η^2^_*p*_ = 0.24. There was also a significant difference between the three utterance content categories when combined across genre, *F*_(1.50, 35.87)_ = 17.13, *p* < 0.001, η^2^_*p*_ = 0.42. Collapsing across book genre, the frequency of story-specific utterances (*M* = 4.58, *SD* = 2.79) was significantly greater than the frequency of both physical descriptions (*M* = 1.74, *SD* = 1.54), *t*_(24)_ = 4.83, *p* < 0.001, and natural facts (*M* = 2.50, *SD* = 1.61), *t*_(24)_ = 3.77, *p* = 0.001. The frequencies of natural facts and of physical descriptions were not significantly different when correcting for multiple comparisons, *t*_(24)_ = 2.32, *p* = 0.029.

**Figure 2:**
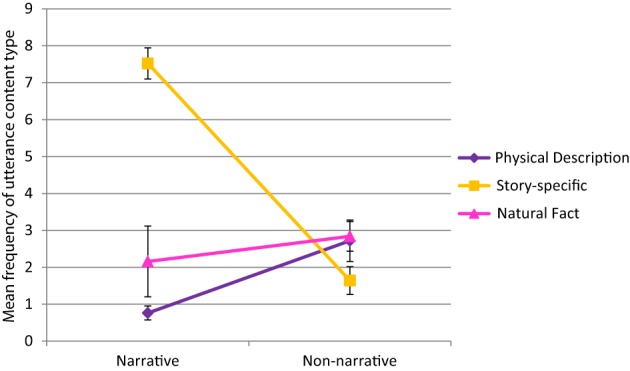
**Mean frequency (±SE) of physical description, story-specific, and natural fact statements across narrative and non-narrative book sharing**.

Turning to the main results of interest, there was a significant genre by content interaction, *F*_(1.53, 36.60)_ = 35.85, *p* < 0.001, η^2^_*p*_ = 0.60. Figure 2 displays the results of this interaction. We found that mothers' use of physical descriptions was significantly greater during non-narrative book sharing than during narrative book sharing, *t*_(24)_ = 3.44, *p* = 0.002. Mothers' produced significantly more story-specific utterances during narrative book sharing than during non-narrative book sharing, *t*_(24)_ = 6.17, *p* < 0.001. Of most interest, there was no significant difference across genres in the frequency of natural facts, *p* = 0.165.

#### Proportion

The patterns of significance for the main effects and interaction were the same, regardless of whether the results were analyzed by frequency or proportion of total maternal utterances. It is noted for the reader, that although the proportion of natural facts was higher during non-narrative book sharing than narrative book sharing, the difference was not significant when corrected for multiple comparisons.

## Discussion

The influence of placing animal characters in a narrative or non-narrative picture-book context on mothers' use of certain types of pedagogical language was investigated in the present study. Because the target animals did not differ between the two genres, and each book presented the six target animals' labels as the only text, the key difference was in whether the animals were presented within illustrations depicting a story (narrative condition) or on a blank page (non-narrative condition). There were no differences across genres in terms of the frequency or proportion of total utterances that were about the animals in the books (i.e., animal-referring). Dividing these animal-referring utterances into generic, specific, and labeling subtypes, we found that specific utterances were significantly more common during narrative book sharing. Surprisingly, however, the two genres did not differ in terms of the frequency or proportion of maternal generic utterances that they engendered. When analyzed by proportion, mothers' use of specific utterances did not differ across the two genres, whereas a greater proportion of utterances were labels during non-narrative book sharing. Thus, the key difference between the two genres was in their elicitation of specific and labeling utterances, not, as one might have predicted based on previous studies, generic utterances (e.g., Gelman et al., 2013).

When looking beyond the structure of mothers' utterances, we found differences in the types of content mothers were delivering during narrative and non-narrative book sharing. Whereas the non-narrative books encouraged more physical descriptions, the narratives, somewhat expectedly, encouraged more story-specific utterances that described states of the animals and animal-related actions on the page. The books did not differ, however, in their propensity to encourage natural facts about the animals. Like the non-significant generic finding, this suggests that mothers do not necessarily take a more pedagogical stance during non-narrative book sharing. The natural fact category comprised utterances that provided information about animals' diet, habit, and behaviors. Note that the generic and natural fact categories were neither completely overlapping nor mutually-exclusive. Although natural facts and generic knowledge may be seemingly synonymous, we found that mothers presented natural facts using specific noun phrases on several occasions (e.g., “He (badger) uses his sharp claws and goes dig, dig, dig.”). As such, the utterance framing and utterance content codings provide complementary, yet unique information.

The present findings add to a growing body of research indicating that contextual influences, and particularly the format of presentation of animals and objects, can influence the extent to which they are construed and spoken about as individuals or members of a kind (Gelman and Tardif, 1998; Gelman et al., 2005, 2013). Language referencing individuals and kinds differs not only across settings (e.g., objects vs. pictures; Gelman et al., 2005), but also within the book sharing setting, when the nature of the book is manipulated.

### Thinking and talking about individuals and kinds

What do these cross-genre differences in utterance framing and content tell us about how animals are construed in narrative and non-narrative contexts? The narratives, which present animals engaging in unique, intentional activities, appeared to prime mothers to think about the animals mostly as individuals, as reflected in the higher frequency and proportion of specific utterances. In other words, given a depicted event, mothers used specific language to describe it. But generic, kind-referring talk was also notably present during narrative book sharing. What is more intriguing, perhaps, is what mothers did with the non-narrative books. In a sense, one should have free rein to discuss whatever one likes about a picture of an animal on a page, as there is no need to convey a visual narrative to the child. Mothers provided a mix of physical descriptions, natural facts, and even some story-specific utterances during non-narrative book sharing. They did not appear, however, to treat the animals in the non-narratives as any more representational of their kind than those in the narratives.

Gelman et al. (2005) have argued that labels are more representational of kinds than specific utterances, but we would argue that labels only serve to place an individual in a category and offer little kind-based information if they are not followed with a generic statement. And, our finding that generics did not differ across genres suggests that mothers did not necessarily view animals in the non-narrative as more representational than those in the narrative. If pedagogical contexts do indeed encourage the communication of generic information, as suggested by recent studies (Gelman et al., 2005; Butler and Markman, 2012) and Csibra and Gergely's (2009) theory of natural pedagogy, then one could conclude from the present findings that mothers were not behaving more pedagogically during non-narrative book sharing than during narrative book sharing.

It is difficult to provide a conclusive answer to the question of whether animals were construed as individuals or as members of a kind in the narrative and non-narrative books by mothers, but, for now, the answer seems to be “both.” Within each type of book, mothers shifted from describing an animal as a specific individual (e.g., narrative: “He's going to sleep in there, too”; non-narrative: “He kind of looks like a dog”) to describing it as a member of a kind (e.g., narrative: “Squirrels eat nuts, don't they?”; non-narrative: “Hyenas laugh, hahaha”). Thus, mothers appear to flexibly consider a single animal as both an individual and as an exemplar when discussing it with their children.

It is worth acknowledging that, relative to other types of talk, the proportion of generics observed in the present study (4–5%) might be considered by readers to be fairly low. However, our observed proportion is similar to that of a previous study with a similar age group (19- to 23-month-olds) (Gelman and Tardif, 1998), in which 4.67% of maternal utterances included generic noun phrases during book sharing. Relative to other contexts (e.g., toy play) and subject matter (e.g., artifacts), the authors found that mothers' use of generic noun phrases was greatest during book sharing about animals. Thus, it would appear that a proportion around 4–5% may be quite typical of book sharing interactions between parents and toddlers. It is likely that as children's comprehension of generic language increases, so too does the amount of generic language they receive in their input. Future research may investigate whether the observed pattern, in which mothers' use of generic language is equivalent across narrative and non-narrative books, holds with slightly older children.

### Learning from narratives and non-narratives

Together with our prior finding, using the same data set, that mothers' talk was more complex during narrative book sharing, with more text-to-life references, mental state terms, and non-present tenses, compared to non-narrative (didactic) book sharing (Nyhout and O'Neill, 2013), the present findings suggest that narratives do indeed provide ample stimulus for abstract and pedagogical types of talk, such as generic language and natural facts, previously assumed to fall more within the domain of informational books.

Anecdotally, many parents who have participated in studies in our lab have remarked that storybooks are “just for fun.” Comments on websites such as Amazon frequently reflect this sentiment. This view may be widely held, given media reports on declining picture book sales in favor of chapter books and more “educational” books (Bosman, 2010). Our findings suggest however that, when sharing wordless books with their mothers, children are exposed to an equal amount of generic, factual information when the book is a narrative as when it is a non-narrative.

Increasingly, researchers and educators are emphasizing the need for children to be exposed to more informational texts both at home and in the classroom (e.g., Reese and Harris, 1997; Duke, 2000, 2003; Goodwin and Miller, 2013). A common argument is that as children shift from *learning to read* to *reading to learn*, they must have access to books that provide “information about the natural and social world” (Duke, 2003, p. 1). Proponents of increasing children's exposure to informational books generally acknowledge that narratives do have their place, but not as potential sources of world knowledge. A key problem that we see with these views is in the strict dichotomization of narrative and informational texts. As demonstrated in the present study and as found in a content analysis of children's books (Gelman et al., 2013), narratives can and do provide generic information about the natural world. Given that our narrative and non-narrative books fostered equal numbers of generic utterances, a key consideration for parents hoping to expose their children to more educational materials should be enjoyment. Those books that parents and children find more enjoyable should maintain attention and potentially facilitate greater learning.

The focus of future investigations should now turn to how and what children learn from narrative and non-narrative books, in both controlled experiments and in more open-ended interactions with caretakers. Previous findings are somewhat mixed in terms of whether children are able to learn factual information from fiction (Fazio and Marsh, 2008; Richert et al., 2009; Richert and Smith, 2011). Most relevant to the present study, perhaps, are the mentioned findings by Ganea et al. (2011), who found that children were able to learn and generalize from both factual and intentional picture book formats. How the two types of books, narrative and non-narrative, may differentially support learning should be a focus of future research. Although our narrative and non-narrative books promoted a relatively equal proportion of generic language, it is unknown to what extent the interactions around the two types of books may have fostered short- and long-term *learning* of information about the featured animals.

### Toddlers' reasoning about animals and objects in books

Three important questions about children's comprehension and reasoning for future research arise from our observations of mothers' shifting use of specific and generic language use during book sharing.

1. *To what extent do children under 2 years of age distinguish between syntax referring to individuals and syntax referring to kinds?* Gelman and Raman (2003) found that 2-year-olds were able to distinguish between questions referring to individuals (e.g., “What color are the dogs?”) and kinds (e.g., “What color are dogs?”) It is unknown how children younger than 24 months may interpret these different sentences. What do children of the age in our study think “This elephant loves peanuts” refers to: the elephant on the page, or all elephants?
2. *Do children construe the animals they encounter in books as individuals, members of a kind, or both?* Because mothers switched between specific and generic utterances frequently, and because there is currently no evidence that children under 2 years of age can distinguish between generic and non-generic syntax, it is worth considering how children may spontaneously conceive of the animals and objects they encounter in books and how this may influence their learning. Intriguingly, it seems that children have a natural propensity to consider and express generics from an early age. Preschoolers who had not been exposed to conventional language (deaf children of hearing parents) produced generics in their home sign system (Goldin-Meadow et al., 2005). However, we do not know what tendency young children have to consider animals in books and pictures and whether narrative context may influence this. It would be of interest to investigate toddlers' and preschoolers' comments about animals in the two types of books when they are asked by a parent or experimenter to provide them.
3. *Can toddlers shift between representing the same entity as both an individual and as an exemplar?* By the age of three, children are capable of a relevant representational ability: dual representation, the ability to represent a symbol both as standing for something else and as a concrete entity itself (DeLoache, 2000). In the case of the toddlers in our study, the question is whether they can consider the animals in our books both as individual characters carrying out unique activities and as exemplars of a kind. The 3- and 4-year-old children in Ganea et al.'s (2011) study were likely able to represent the animals in the intentional, narrative condition as individual characters and as members of a kind, evidenced by their ability to generalize the information learned. It is unknown whether the toddlers in our study were able to consider the unique activities of the animals on the page (e.g., a squirrel sneaking into a bear's den to keep warm), while also making predictions about the kind-relevant activities the animals must engage in (e.g., collecting and hiding nuts). Many of the children in the present study frequently produced animal sounds for known animals in the books, both spontaneously and when requested by mothers. This suggests that they readily considered the animals on the pages as exemplars of a kind.

## Conclusion

Much of the information we possess about the world comes from the books we read, the films we watch, and the people we converse with. Although non-fiction books and documentary films may first come to mind when one thinks about the genres of media that are likely to provide natural facts about the world, the present findings suggest that both narrative and non-narrative children's picture books stimulate such pedagogical talk from mothers. While the narrative books promoted more references to individual characters, the non-narrative books elicited more instances of labels. Surprisingly, the two types of books encouraged similar amounts of generic talk about kinds of animals and talk about natural facts. Based on these findings, we leave the reader with one final piece of generic information: picture book stories aren't just for fun; they're for learning, too.

### Conflict of interest statement

The authors declare that the research was conducted in the absence of any commercial or financial relationships that could be construed as a potential conflict of interest.
